# Seroepidemiological Survey of Anti-*Toxoplasma gondii* and Anti-*Neospora caninum* Antibodies in Domestic Cats (*Felis catus*) in Rolim de Moura, State of Rondônia, North Brazil

**DOI:** 10.3390/tropicalmed8040220

**Published:** 2023-04-10

**Authors:** Ana Luzia Peixoto Silva, Estefany Ferreira Lima, Geraldo Moreira Silva Filho, Larissa Claudino Ferreira, Beatriz de Andrade Campos, Ividy Bison, Arthur Willian de Lima Brasil, Roberta Nunes Parentoni, Thais Ferreira Feitosa, Vinícius Longo Ribeiro Vilela

**Affiliations:** 1 Departamento de Medicina Veterinária, Instituto Federal da Paraíba—IFPB, Sousa 58807-630, Paraíba, Brazil; 2 Programa de Pós-Graduação em Ciência e Saúde Animal, Universidade Federal de Campina Grande—UFCG, Patos 58708-110, Paraíba, Brazil; 3 Departamento de Medicina Veterinária, Universidade Federal de Rondônia—UNIR, Rolim de Moura 76940-000, Rondônia, Brazil; 4 Departamento de Morfologia, Universidade da Paraíba, João Pessoa 58059-900, Paraíba, Brazil

**Keywords:** cats, toxoplasmosis, antibodies, zoonosis

## Abstract

Epidemiological studies on infections by *Toxoplasma gondii* and *Neospora caninum* in cats in the North Region of Brazil are scarce. We intended to assess the seroprevalence in cats of anti-*T. gondii* and anti-*N. caninum* antibodies, as well as the associated risk factors that may lead them to contract these infections in Rolim de Moura, state of Rondônia, North Brazil. For this, blood serum samples of 100 cats from different regions of the city were evaluated. To assess possible factors associated with infections, epidemiological questionnaires were applied to tutors. The Immunofluorescence Antibody Test (IFAT) was performed for anti-*T. gondii* (cutoff 1:16) and anti-*N. caninum* (cutoff 1:50) antibodies. After identifying the positive samples, antibody titration was performed. The results showed the prevalence of 26% (26/100) of anti-*T. gondii* antibodies, with titration varying between 1:16 to 1:8192. There were no factors associated with the prevalence of anti-*T. gondii* antibodies in the multivariate analysis in this study. There was no occurrence of seropositive cats for anti-*N. caninum*. It was concluded that there was a high prevalence of anti-*T. gondii* antibodies in cats in Rolim de Moura, state of Rondônia, North Brazil. However, the evaluated animals did not present anti-*N. caninum* antibodies. Therefore, knowing that *T. gondii* has different transmission forms, we emphasize the importance of spreading more information to the population about cat’s relevance in the *T. gondii* life cycle and how to avoid the parasite transmission and proliferation.

## 1. Introduction

The coexistence between humans and animals began at the advent of society and has since provided several benefits for both species; in fact, it is evident that this relationship played an important role in human evolution. Records show that cat domestication dates back 4000 years and occurred in a commensal manner [1]. Consequently, with the population growth trend and societal development associated with demographic and social changes, there was also an increase in the animal companion population linked to both an increase in the living standard and a decrease in family size [2]. However, it is known that these animals are affected by different types of pathogens potentially transmissible to humans, thus bringing risks to public health.

Among the agents that affect domestic cats (*Felis catus)*, *Toxoplasma gondii* is one of the most researched and relevant, since cats are significant for the maintenance of its life cycle [3,4,5]. It is known that the genus *Toxoplasma* contains only one species, and can be grouped into genotypes I, II, III and XII, and haplotypes X and A, in which some are restricted to wild animals [6]. It is an obligate intracellular protozoan parasite, which is found globally and can infect almost all warm-blooded animals [7]. Felines are the definitive hosts of this parasite, in which their sexual reproduction occurs, promoting the environmental oocyst excretion, spreading the infection to rodents and a wide range of animals [8]. All hosts can become infected through different routes, such as food and water contaminated with sporulated oocysts, ingestion of tissues containing cysts, congenital transmission, blood transfusion, and organ transplantation [6].

*Neospora caninum*, like *T. gondii*, is an intracellular coccidian parasite that affects several species of animals, of which Canines are its definitive hosts; the parasite does not affect humans [9]. Cats are intermediate hosts, in which the close contact with dogs can promote high seropositivity rates of IgM anti-*N. caninum.* However, it has been observed that only a small number of cats previously infected with *N. caninum* persisted with antibodies for a long time [10].

Up to one third of the world’s human population is infected with *T. gondii*; however, most infections are asymptomatic [11]. Latent infection promotes cyst formation in different organs, particularly cardiac and skeletal muscle, brain parenchyma, and the retina [12]. Clinically, it causes myocarditis, lymphadenopathy, hepatosplenomegaly, brain abscess, meningitis, chorioretinitis, pneumonitis, hepatitis, and pancytopenia [13]. In clinical investigations of feline toxoplasmosis, it was demonstrated that depending on the exposed strain, affected cats present symptoms of apathy, hyporexia, bristly hair, nasal secretion, ocular secretion, and diarrhea, and in acute cases, death [4,14]. In addition, neosporosis causes a fatal neurological disease in dogs, and it is very important for the cattle production sector, since it causes high rates of miscarriages and stillbirths [15]. In the Feline species, however, there are reports that *N. caninum* can promote transplacental transmission during acute and chronic infections and cause myositis and neurological diseases, such as encephalitis [16,17,18].

In a meta-analysis to assess the prevalence of *T. gondii* in cats in Brazil, Lugoch, Noro, and Andrade [19] observed a seroprevalence of 35.9% in cats, where 50.5% of the cats came from the North, Northeast, and Midwest. In the state of Roraima, North Brazil, Gomes et al. [20] described a prevalence of anti-*T. gondii* and anti-*N. caninum* antibodies in 65.63% (21/32) and 15.62% (5/32), respectively, demonstrating that despite the small sample number, there were important prevalence rates for these antibodies. Therefore, due to the few epidemiological studies on infections by these parasites in the North Region of Brazil, the aim of this research was to evaluate the seroprevalence for anti-*T. gondii* and anti-*N. caninum* antibodies in cats in Rolim de Moura, state of Rondônia, and the associated risk factors associated with these infections.

## 2. Materials and Methods

### 2.1. Geographic Characterization of the Study Site

The sample collections were carried out in the city of Rolim de Moura, state of Rondônia. Located in the western Amazon, in the North Region of Brazil, it is characterized by a hot humid equatorial climate, with a long rainy period, an annual rainfall average of 1896.5 mm, and only one dry quarter between June and August [21]. In this region, most of the year has a favorable climate for the occurrence of the *T. gondii* life cycle [22], and consequentely *N. caninum*. After environmental excretion into ideal conditions of temperature (average of 20 °C), humidity (average of 65%), pressure, and oxygenation, oocysts take from one to five days to become infectious [23].

### 2.2. Sample Population

It was a cross-sectional study, and the sampling was designed to determine the prevalence of positive cats. To determine the minimum sample number to be used, a simple random sampling was applied, as recommended by Thrusfield [24]:

$$n=\frac{z^{2}\cdot P\;\left(1-P\right)}{d^{2}}$$

where:

*n*: number of cats selected;

z: normal distribution value at the confidence level;

P: expected prevalence;

d: margin of error.

Sampling of cats was carried out with a confidence level of 95%, expected prevalence of 50% and margin of error of 10%.

This was adjusted for the local population, using the formula:

$$n_{ajus}=\frac{N\;\cdot \;n}{N+n}$$

where:

*n_ajus_*: final number of cats selected;

n: number of cats selected;

N: population of cats that exist.

We adjusted the sample population size considering the cat human ratio of 18.98 described by Canatto et al. [25]. The estimated population of Rolim de Moura, state of Rondônia, was 55,748 people [26], so, it was estimatedthat there was a population of 2937 cats. Thus, the minimum number of animals to be used in the study was 94; however, samples of 100 cats were collected. The geographic locations of the houses visited were also collected.

### 2.3. Serological Analyses

To obtain the samples, a direct and active random criteria search without selection was carried out for choosing the animals. After the consent of the tutors, blood samples were collected by jugular vein puncturing, centrifuged to obtain serum, stored at −20 °C, and sent for processing and analysis at the Laboratory of Immunology and Infectious Diseases, Federal Institute of Paraíba (IFPB), Sousa campus. The serum was analyzed by Immunofluorescence Antibody Test (IFAT). The strains used for the diagnosis were ME-49 (*T. gondii)* and Nc-1 (*N. caninum*), with tachyzoites fixed in slides, at cutoffs of 1:16 and 1:50, respectively [27,28]. Anti-cat IgG conjugate (SIGMA, St. Louis, MO, USA) was used. Samples considered positive were subjected to two-fold sequential dilutions to determine the antibody titration.

### 2.4. Epidemiological Questionnaires

An epidemiological questionnaire was applied to the tutors in order to collect information related to the breed, sex, and age. Data related to environmental management, and contact with other domestic animals (other cats, cattle, sheep, pigs, poultry, dogs, and equids) and wild animals were also obtained, along with data about food management, considering the type of food, and food storage. Lastly, data were obtained regarding reproductive and health characteristics, such as occurrences of abortions, number of deliveries, deworming (frequency and active ingredient of the deworming agent), vaccination and current or past diseases.

### 2.5. Statistical Analysis

Data regarding the factors associated with the presence of anti-*T. gondii* and anti-*N. caninum* antibodies came from epidemiological questionnaires. In the univariate analysis, each independent variable was cross-correlated with the dependent variable (seropositivity), and those with a *p*-value ≤ 0.2, according to the chi-square test or Fisher’s exact test, were selected for multivariate analysis using robust Poisson regression. In order to verify some collinearity between the data, a correlation test was applied. If the correlation coefficient was greater than 0.9, one of the variables was eliminated using biological plausibility criteria. To verify the model fit, the chi-square parameters and the Omnibus test were used. Multivariate analysis was performed at the 5% level in the SPSS 23.0 software.

## 3. Results

**Figure 1:**
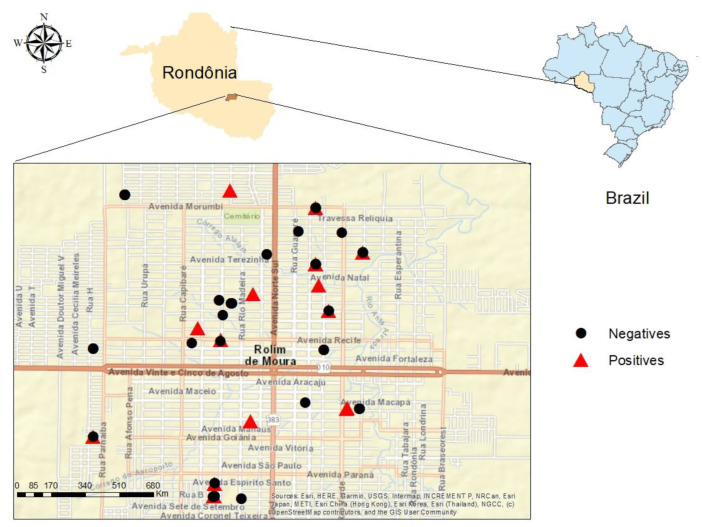
Geographical location of the municipality of Rolim de Moura, state of Rondônia, Brazil. Place of origin of cats and spatial distribution between seropositive and seronegative for anti-*T. gondii* antibodies.

Among the 100 cats evaluated, anti-*T. gondii* antibodies were detected in 26% (26/100), with 57.7% (15/26) being females and 42.3% (11/26) being males (*p* > 0.2). The geographic location of the houses and the occurrence of outbreaks of seropositive animals for *T. gondii* are described in Figure 1.

A variation was observed in the anti-*T. gondii* titration, between 1:16 and 1:8192 (Table 1).

There was no occurrence of seropositive cats for anti-*N. caninum* antibodies in this study.

The variables associated with anti-*T. gondii* antibodies positivity that were significant in the univariate analysis (*p* ≤ 0.2) were: mixed breed animals, no access to treated water, partial maintenance of the animal on a dirt floor, and cleaning and disinfection of the environment fortnightly or monthly (Table 2).

There were no risk factors associated with the prevalence of anti-*T. gondii* antibodies in the multivariate analysis in the present study.

## 4. Discussion

The seroprevalence results of anti-*T. gondii* antibodies found in the present study (26%; 26/100) corroborate data by Souza et al. [29], who in the state of Acre, North Brazil, found a seroprevalence of 24.7% (22/89) in cats, using IFAT. However, this percentage is low when compared to older studies in the region. In the state of Amazonas, positivity was observed in 90.6% (29/32) of the studied cats, with Indirect Hemagglutination (HI) [30]; in Rondônia, 87.3% (55/63), with Modified Agglutination Test (MAT) [31]. However, it is important to attempt the study because of the different accuracy rates among the different diagnostic tests used [32,33].

There was a predominance of animals with high antibody titers, with 76.9% (20/26) of the positive cats with titers between 1:128 and 1:8192 [34]. When analyzing these high IgG titrations, it is assumed that the cats have the infection under control and that they probably excreted oocysts, since they stop excreting them in the feces when the peak of IgM antibody production occurs [35]. Thus, when observing the wide geographic distribution of seropositive animals, it is possible for wide transmission foci dissemination to occur, mainly in public squares, leisure, and cultivation areas [36,37,38]. It is also known that cats shed oocysts in the primary infection and can shed oocysts again after parasitic reinfection, in rates 30% less than the first infection [36], which raises public health concerns.

In the present study, there was no occurrence of seropositive animals for anti-*N. caninum*. However, in North Brazil, in the state of Roraima, Gomes et al. [20] found seropositivity for anti-*N. caninum* in 15.62% (5/32) of the evaluated cats. Similar to the results obtained in the present study, Feitosa et al. [39], in the state of Paraíba, Northeastern Brazil, in research with domestic and stray cats did not find seropositive animals for anti-*N. caninum*. Cats are more resistant and less exposed to *N. caninum*, with a very low or absent prevalence of antibodies being observed [40,41]. There is still little information on the pathogenesis of neosporosis in cats [10].

Mixed breed cats had higher prevalence of anti-*T. gondii* antibodies (*p* ≤ 0.2). It is important to emphasize that this association is not biologically significant. This occurs because, in Brazil, they receive less sanitary management when compared to thoroughbred cats, making them more vulnerable to infections [29].

The higher seropositivity observed in animals kept in ground environments (*p* ≤ 0.2) and fortnightly/monthly cleaning (*p* = 0.2) can occur due to the presence of high humidity, that favors the high oocyst survival capacity in tropical areas [42].

Oocyst transmission via untreated water, according to Silva et al. [43], is responsible for toxoplasmosis outbreaks and accordingly, the present study also demonstrates that animals without access to treated water had higher prevalence for anti-*T. gondii* antibodies (*p* ≤ 0.2). This can be an important infection route for *T. gondii* infection in the studied region, once it was reported in cases of feline [44] and human toxoplasmosis outbreaks in other regions of Brazil [45,46]. It is also important to highlight that *T. gondii* infection is more likely favored mainly due to inadequate hygiene, inadequate disposal of cat litter, and especially when one has the habit of gardening and agriculture [47,48].

## 5. Conclusions

It was concluded that there was a high prevalence of anti-*T. gondii* antibodies in cats in Rolim de Moura, state of Rondônia, North Brazil. However, the evaluated animals did not present anti-*N. caninum* antibodies. Although risk factors for *T. gondii* infections were not obtained, there was higher positivity in animals which were mixed breed, had access to untreated water, and lived on the ground floor that was cleaned and disinfected every fortnight or month.

## Figures and Tables

**Table 1:** Distribution of antibody titration for anti-*T. gondii* in seropositive cats in the municipality of Rolim de Moura, state of Rondônia, North Brazil.

Titration	Number of Animals	Percentage
16	4/26	15.4%
32	1/26	3.8%
64	1/26	3.8%
128	1/26	3.8%
256	4/26	15.4%
512	5/26	19.2%
1024	4/26	15.4%
2048	3/26	11.6%
4096	2/26	7.7%
8192	1/26	3.8%
Total	26/26	100%

**Table 2:** Univariate analysis of risk factors associated with anti-*Toxoplasma gondii* antibodies positivity in cats (*n* = 100) in the state of Rondônia, North Brazil.

Variable	Category	Total Cats	*n* of Positives	*p*
Breed	Thoroughbred	23	3 (13.0)	0.131
	Mixed	77	22 (28.6)	
Access to treated water	No	7	4 (57.1)	0.042
	Yes	93	21 (22.6)	
Where the animal is kept	Cement	38	8 (21.1)	0.186
	Ground	1	1 (100)	
	Both	61	16 (26.2)	
Environmental cleaning and disinfection	Daily	72	16 (22.2)	0.2
	Weekly	25	7 (28.0)	
	Fortnightly/Monthly	3	2 (66.7)	

## Data Availability

Not applicable.
